# Thermally Treated Berberine-Loaded SA/PVA/PEO Electrospun Microfiber Membranes for Antibacterial Wound Dressings

**DOI:** 10.3390/polym14214473

**Published:** 2022-10-22

**Authors:** Jishu Zhang, Yonggang Li, Huawei Wu, Chunhong Wang, Kushairi Mohd Salleh, Hongchang Li, Sarani Zakaria

**Affiliations:** 1 School of Textile, Garment & Design, Changshu Institute of Technology, Changshu 215500, China; 2 School of Textile Science and Engineering, Tiangong University, Tianjin 300387, China; 3 College of Engineering and Technology, Jiyang College of Zhejiang A & F University, Shaoxing 312000, China; 4 Bioresource Technology Division, School of Industrial Technology, Universiti Sains Malaysia, Penang 11800, Malaysia; 5 Renewable Biomass Transformation Cluster, School of Industrial Technology, Universiti Sains Malaysia, Penang 11800, Malaysia; 6 Bioresources and Biorefinery Laboratory, Faculty of Science and Technology, Universiti Kebangsaan Malaysia, Bangi 43600, Malaysia

**Keywords:** calcium chloride, fracture strength, Trition X-100, water dissolution resistance, green product

## Abstract

This study aimed to develop a safe and advanced antibacterial material of electrospun microfiber membranes (MFMs) for wound dressings. Combinations of several materials were investigated; thermal treatment and electrospinning techniques were used to form the best quality of MFMs to suit its end applications. By comparing the fiber morphology, diameter changes, and fracture strength, the suitable ratio of raw materials and thermal treatment were obtained before and after adding Trition X-100 as a surfactant for MFMs of sodium alginate/polyvinyl alcohol/polyethylene oxide (SA/PVA/PEO). The electrospinning solution was mixed with berberine as an antibacterial substance; meanwhile, calcium chloride (CaCl_2_) was used as the crosslinking agent. The antibacterial properties, water dissolution resistance, water content, and fracture strength were thoroughly investigated. The results showed that the antibacterial rates of MFMs with different mass fractions of berberine (0, 3, and 5 wt.%) to *Escherichia coli* (*E. coli*) were 14.7, 92.9, and 97.2%, respectively. The moisture content and fracture strength of MFMs containing 5 wt.% berberine were 72.0% and 7.8 MPa, respectively. In addition, the produced MFMs embodied great water dissolution resistance. Berberine-loaded SA/PVA/PEO MFMs could potentially serve as an antibacterial wound dressing substrate with low cost and small side effects.

## 1. Introduction

With the growth of the geriatric population, chronic wound care problems are increasingly serious. Traditional wound dressings such as bandages, cotton wool, and gauzes limit swelling capacity, have problems with moisture vapor permeability, and promote dryness and adherence to the wound surface, causing considerable pain upon removal and secondary damage [1,2]. A good wound dressing must possess antibacterial activity, a thin layer, and high mechanical strength, as well as maintaining the wound’s moist environment, allowing gas exchange, being non-adherent, and acting as a barrier to avoid pathogen penetration [3,4,5]. Therefore, developing high-performance antibacterial wound dressings has an important significance in the application. For this reason, microfiber membranes (MFMs) are good candidates to fulfill these requirements. With high surface area, substantial mechanical strength, and proper materials selection, developing a high-performance antibacterial wound dressing is viable. In the exploration of potential MFMs, a profound understanding of techniques and materials must first be tackled.

Microfibers can be prepared via various processing techniques such as self-assembly, template synthesis, phase separation, electrospinning, etc. [6,7]. Among these, the most direct and simple method is the electrospinning process. Electrospinning process is widely used in the preparation of wound dressings due to its simple operation and low cost, and it does not change the characteristics of the solution itself [8]. The obtained microfibers usually possess larger specific surface area, which is good for better contiguity with the wound, and smaller pore size to prevent instant dryness [9,10,11]. In addition, MFMs prepared by electrospinning have high porosity and good air permeability, which can provide a moist environment on the wound surface and facilitate cell respiration [12,13].

The electrostatic spinning device comprises three parts: a high-voltage power supply, a syringe pump with a capillary syringe, and a collection device. The syringe needle is connected with the polymer to the anode end of the high voltage power supply, and then the voltage is turned on to charge the polymer. In the case of an external electric field force, the charged polymer droplets will be stretched and deformed, forming a “Taylor cone” through the combined action of electric field force and surface tension. As the electric field strength increases and the voltage reaches a certain threshold, the charge repulsion on the polymer surface breaks the surface tension and viscosity, forming a jet. Finally, the jet falls on the receiving plate to form the MFM [14,15].

Currently, wound dressings are commonly divided into natural and synthetic materials. Wound dressings made from bioresource materials are more favorable due to renewability, biocompatibility, and biodegradability. Extracted sodium alginate (SA) from algae is among the bioresources often employed in wound dressings applications [16,17,18]. Alginate is formed by β-D-mannuronic acid (unit M) and α-L-guluronic acid (unit G), and the order of the units determines the physical and chemical properties [19]. The main products of SA wound dressings are hydrogel, film, and microfiber [20,21,22]. SA has been shown to have the capability to imitate the structure of human extracellular matrix (ECM) while steering cells’ behavior and functionality [23,24]. Microfibers are soft and porous, which can effectively absorb wound exudates, making them suitable carriers for drug delivery applications. SA microfiber wound dressings prepared by electrospinning are non-toxic and difficult to adhere to the skin, have good air permeability, and exudate absorption can quickly stop bleeding [25,26]. Hence, the availability and renewability of SA, combined with the low production cost of the electrospinning process, can provide a substitute for the long reigning synthetic materials in wound dressing applications.

In an aqueous solution, the macromolecular chain of SA is a rigid and worm-like-structure, which eventually makes the electrospinning process become very challenging. Thus, a material with macromolecular chains of strong hydrogen bonds and good entanglement such as polyvinyl alcohol (PVA) is required to overcome this. With the addition of PVA to the SA solution, the effect of electrostatic spinning on the formation of microfibers has substantially improved [27,28,29]. Nevertheless, with the increase of SA content, macromolecules of SA are prone to break the hydrogen bond of PVA macromolecules, resulting in a considerable decline of mechanical properties [30]. Hence, proper SA to PVA ratios must be scrutinized.

Polyethylene oxide (PEO) is a water-soluble polymer with better entanglement than PVA, non-toxic, and easy to degrade [31]. PEO can be used to adjust the electrical conductivity and MFMs morphology of the SA electrospinning solution [32]. In addition, PEO is a thermoplastic polymer with a melting point of about 65 °C, while the melting points of both SA and PVA are above 150 °C [33,34,35]. The PEO thermal behavior implies improved molten adhesion for MFMs and subsequently affects the mechanical properties of MFMs.

With the electrospinning process, a solution with high surface tension and high electrical conductivity could decrease the efficiency of the process and the quality of the MFMs produced. Hence, to solve this predicament, Triton X-100 is a perfect surfactant to be mixed with the electrospinning solution, as it can substantially decrease electrical conductivity and surface tension [36,37]. Triton X-100 also exemplifies low cytotoxicity, thus making it suitable for medical applications. Furthermore, the addition of Triton X-100 in the electrospinning solution enhances SA efficiency while ensuring the mechanical properties of MFMs [38].

Good antibacterial property is necessary for wound dressings [39]. An antibacterial substance in the MFMs will extend shelf life by protecting it from being colonized by bacteria and by exhibiting an antibacterial property. Berberine is a quaternary ammonium alkaloid isolated from Coptis chinensis that possesses an antibacterial property. It has significant bacteriostatic effects on various pathogenic microorganisms such as Staphylococcus aureus, Escherichia coli, and Pseudomonas aeruginosa [40,41]. In contemplating obtaining antibacterial properties, traditional wound dressings have been developed by combining dressing materials with antibiotics and with metal ions with antibacterial properties, such as silver compounds and nano zinc [42,43]. However, excessive use of antibiotics often occurs, which causes drug resistance in bacteria and causes side effects to other tissues and organs. Furthermore, concerns have been expressed about the development of bacterial resistance to metals ions. Additionally, most metal ions and particles are heavy metals, and they are potentially harmful to human organs [44,45,46]. Dissimilarly, berberine has fewer side effects compared with antibiotics and metal antibacterial particles. It does not make bacteria resistant to drugs, nor does it carry the risk of heavy metals. Nonetheless, the incorporation of berberine in the MFMs’ network has its downside. Based on the proposed materials, the produced MFMs are naturally prone to rapid condensation and dissolve in water, restricting their use in wound dressing applications [47]. Hence, crosslinking is one of the simplest approaches to reduce the solubility [48,49,50].

In this study, SA, PVA, PEO, and Trition X-100 were used as raw materials to produce MFMs via the electrospinning process. The fiber morphology, diameter changes, and fracture strength of SA/PVA/PEO MFMs before and after adding Trition X-100 were investigated. Suitable ratios of each raw material and treatment temperatures were examined. On this basis, different amounts of berberine as an antibacterial agent were added to the electrospinning solution to prepare the MFMs. The produced MFMs have low production cost, good antibacterial property, and small side effects. The produced MFMs could prevent harm to the human body caused by conventional metal-based antibacterial substances and resolve bacterial resistance instigated by antibiotics.

## 2. Materials and Methods

### 2.1. Materials

SA was provided by Tianjin Fuchen Chemical Reagent Factory; PVA (viscosity: 20.5–24.5 cps, pH: 5–7, degree of alcoholysis: 87–89 mole%) was provided by Business Guide-Sha; PEO (molecular weight: 1,000,000) was provided by Guangzhou Lihou Trading Co., Ltd.; Trition X-100 (molecular weight 647W) was provided by Beijing Solaibao Technology Co., Ltd. (Beijing, China); Berberine (active ingredient: 98%) was provided by Xi’an Xiaocao Plant Technology Co., Ltd. (Xi’an China); Beef Cream, Peptone, and Agar were supplied by Beijing Aobaxing Biotechnology Co., Ltd. (Beijing, China); calcium chloride (CaCl_2_) was provided by Tianjin Fengchuan Chemical Reagent Technology Co., Ltd. (Tianjin, China); analytically pure sodium chloride (NaCl) were supplied by Tianjin Chemical Reagent Factory (Tianjin, China).

### 2.2. Equipment

**Figure 1:**
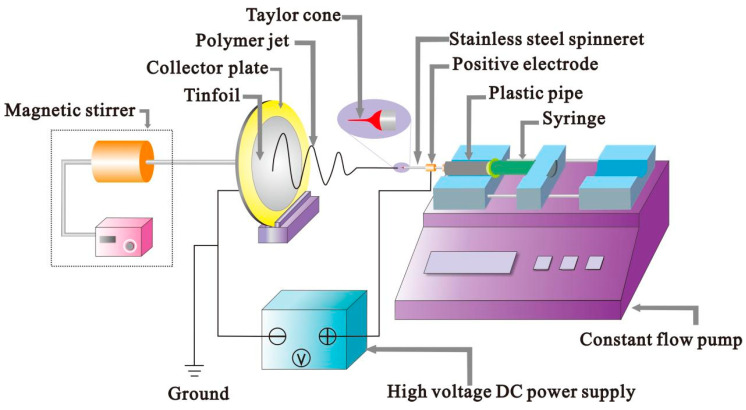
Connection diagram of electrostatic spinning equipment.

The device for preparing microfibers in this experiment was a self-assembled horizontal electrospinning device shown in Figure 1. It mainly consisted of a constant flow pump, collector plate, high voltage DC power supply, syringe, and magnetic stirrer. In the process of electrostatic spinning, the magnetic stirrer drives the collector plate to rotate, producing even microfibers distribution. The collector plate was connected to the high-voltage power supply’s negative pole, and the needle on the transmitter was attached to the positive pole of the power supply. A plexiglass plate was installed outside the generator to avoid the influence of the external environment during the spinning process.

### 2.3. Preparation of SA/PVA/PEO Microfiber Membranes

The SA (2 wt.%), PVA (10 wt.%), and PEO (3 wt.%) solutions were prepared by dissolving them separately in distilled water. SA/PVA/PEO solutions containing two volume ratios (4:3:3 and 5:3:2) of dissolved 2 wt.% SA, 10 wt.% PVA, and 3 wt.% PEO were prepared [38,51]. In addition, SA/PVA electrospinning solutions with volume ratios of 4:6 and 5:5 was prepared for comparison. The prepared solution was loaded into the generator, and the receiving distance was adjusted. Then, the power supply was switched on to adjust the voltage and flow. The receiving disk speed was set to 300 r/min. The spinning time was controlled to 4 h per piece. Trition X-100 (0.5 wt.%) was added to the spinning solutions to prepare the MFMs.

### 2.4. Morphology Observation

The foil containing the MFMs was cut into 10 mm× 5 mm samples, and the morphologies of the electrospinning fibers were observed under TM-1000 SEM (Hitachi, Tokyo, Japan) with ×6000 magnification. The diameter of the electrospinning fiber was measured by Image J.

### 2.5. Preparation and Treatment of Berberine-Loaded SA/PVA/PEO Microfiber Membranes

**Figure 2:**
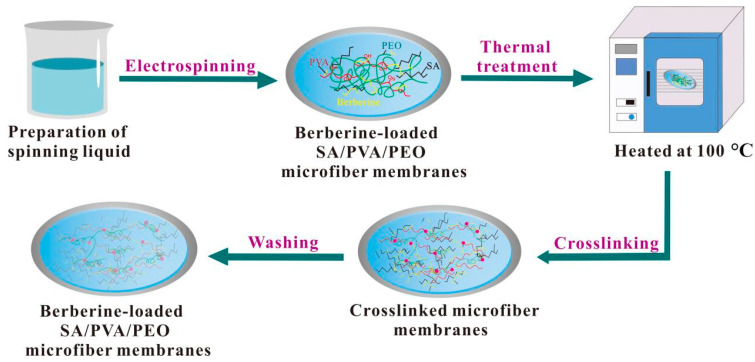
Preparation of berberine-loaded SA/PVA/PEO MFMs.

The whole preparation process was translated in Figure 2. Berberine (mass fractions of 0, 3, and 5 wt.%) was added to the optimal ratio of SA/PVA/PEO solution prior to the electrospinning process to form MFMs. A 16 kV voltage was supplied, and the feed rate for the polymer solution was adjusted to a constant rate of 0.6 mL/h. The magnetic stirrer’s speed and spinning time were set to 200 r/min and 48 h, respectively. The produced MFMs were then thermally treated in an electric blast drying oven at 100 and 120 °C for 2 h. The basic experimental scheme is shown in Table 1. Afterward, the MFMs were immersed in an ethanol solution of 4 wt.% CaCl_2_, which acts as crosslinking exchange and ionic crosslinking media, for 4 h. The crosslinking treatment is used to improve the water dissolution resistance of medical dressings. Finally, the berberine-loaded SA/PVA/PEO MFMs were washed with ethanol solution and dried at 20 °C for 24 h.

### 2.6. Antibacterial Activity Test of Berberine-Loaded SA/PVA/PEO Microfiber Membranes

“GB/T 20944.3-2008-Part 3: Shake flask method” was applied to assess the antimicrobial property of microfiber membranes [52]. The antibacterial rate of SA/PVA/PEO MFMs containing different amounts of berberine to *E. coli* was tested. The circular bacteria were inoculated from cultured *E. coli* (3rd to 10th generation) in test tubes and inoculated on nutrient agar plates and cultured at 37 °C for 18 h. A typical colony was inoculated in 20 mL nutritious broth and was incubated under 130 r/min for 18 h. Then the inoculation suspension was prepared and determined by spectrophotometer; the counted living bacteria were 3 × 105 CFU/mL to 4 × 105 CFU/mL. The MFMs with 0, 3, and 5 wt.% berberine were cut into 5 mm × 5 mm samples and sterilized by ultraviolet irradiation. Samples were then mixed with *E. coli* suspension for shock culture, and the *E. coli* suspension without samples was used as the counter sample. Finally, every mixture mentioned above was diluted for 10^4^, 10^5^, and 10^6^ times, respectively, and placed in a constant temperature biochemical incubator at 37 °C for 24 h. The plate with the appropriate dilution ratio between 30 CFU and 300 CFU was selected.

### 2.7. Water Dissolution Resistance Test of Berberine-Loaded SA/PVA/PEO Microfiber Membranes

According to the liquid absorption rate measurement method, MFMs were cut into 3 cm × 3 cm samples and placed in deionized water [53]. Then the dissolution status of the MFMs before and after crosslinking treatment was observed.

### 2.8. Moisture Content Test of Berberine-Loaded SA/PVA/PEO Microfiber Membranes

Moisture content was measured under the standards of the YY/T 0471.1-2004 [54]. First, the MFMs were dried in an oven at 50 °C for 12 h. Then, they were cut into 1 cm × 1 cm and referred to as G_0_. Next, they were soaked in phosphate buffer saline (PBS) for 2 min and referred to as G_1_. PBS was used to mimic the humoral environment, and MFMs can be biodegradable in phosphate-buffered brine [55,56]. The formulation of PBS buffer is shown in Table 2. The moisture content was calculated by Equation (1).

(1) $${\;S=(G}_{1}^{}-G_{0}^{}{)\;/\;G}_{0}^{}\times 100\%.$$

where S is the moisture content rate (%), G_1_ is the mass of the wet sample (g), and G_0_ is the mass of the dry sample (g).

### 2.9. Fracture Strength Test of Berberine-Loaded SA/PVA/PEO Microfiber Membranes

Fracture testing was performed by Instron 3369 universal strength machine (INSTRON Co., Ltd., Boston, USA). Samples were cut into 90 mm × 10 mm and placed between the jaw with a gauge length of 30 mm. On each sample, three points were selected, and thickness values were measured with a thickness gauge. The fracture strength was calculated by Equation (2).

(2) P=F / (w×d).

where P is the fracture strength (MPa), F is the fracture force (cN), w is the width of the sample (mm), and d is the thickness of the sample (mm).

## 3. Results and Discussion

### 3.1. The Best Ratio of SA/PVA/PEO with a Good Electrostatic Spinning Effect

**Figure 3:**
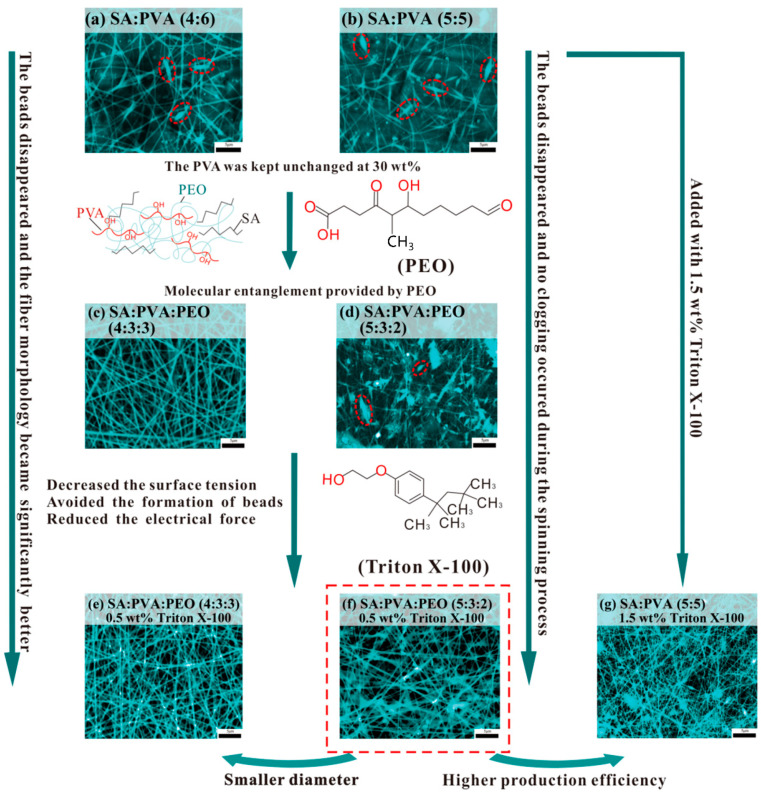
(**a**–**g**) The SEM image (×6000) of seven series of MFMs. (**a**) The SA/PVA MFM in the ratio of 4:6. (**b**) The SA/PVA MFM in the ratio of 5:5. (**c**) The SA/PVA/PEO MFM in the ratio of 4:3:3. (**d**) The SA/PVA/PEO MFM in the ratio of 5:3:2. (**e**) The SA/PVA/PEO MFM in the ratio of 4:3:3 containing 0.5 wt% Triton X-100. (**f**) The SA/PVA/PEO MFM in the ratio of 5:3:2 containing 0.5 wt% Triton X-100. (**g**) The SA/PVA MFM in the ratio of 5:5 containing 1.5 wt.% Triton X-100.

There are apparent differences in morphological structures between MFMs made with SA/PVA and with SA/PVA/PEO. In Figure 3a of SA/PVA 4:6 and Figure 3b of SA-PVA 5:5 MFMs, the formation of beads is visibly observed. Meanwhile, there are no beads formed for SA/PVA/PEO 4:3:3 MFMs, as observed in Figure 3c. The reason is that PEO contributes a better degree of entanglement between macromolecules, thus improving the electrospinning performances of the SA/PVA solution system [57]. Nevertheless, when the ratios between SA/PVA/PEO change to 5:3:2, respectively, very thin microfibers formed with obvious clogging and beads are observed in Figure 3d. With the mass fraction of PEO decreased and that of SA increased, the improvement effect of PEO gradually weakened. Meanwhile, as the mass fraction of SA increased, and the viscosity of the spinning solution gradually decreased, it resulted in insufficient entanglement of macromolecular chains in the spinning solution and discontinuous jet flow [58]. Apparent differences between solutions with and without Triton X-100 are noted. With the addition of 0.5 wt.% Triton X-100 surfactant on SA/PVA/PEO of 4:3:3, microfibers’ structures have improved with a clear reduction of beads, and no clogging is formed, as seen in Figure 3e. For Figure 3f, at the same 0.5 wt.% Triton X-100 surfactant, reduction of beads and fluent spinning are also noticeable for SA:PVA:PEO 5:3:2, in comparison to similar solutions without Triton X-100 in Figure 3d. The reason is that Triton X-100 in solution affected the circularity of beads and improved the homogeneity of fiber corresponding to the decrease in electrical conductivity and the surface tension [59]. In addition, the SA/PVA/PEO 5:3:2 microfibers added to with 0.5 wt.% Triton X-100 of Figure 3f have given better production efficiency than the SA/PVA 5:5 microfibers added to with 1.5 wt.% Triton X-100 of Figure 3g. This has also proven that PEO can effectively promote the preparation of alginate wound dressings by electrospinning.

**Figure 4:**
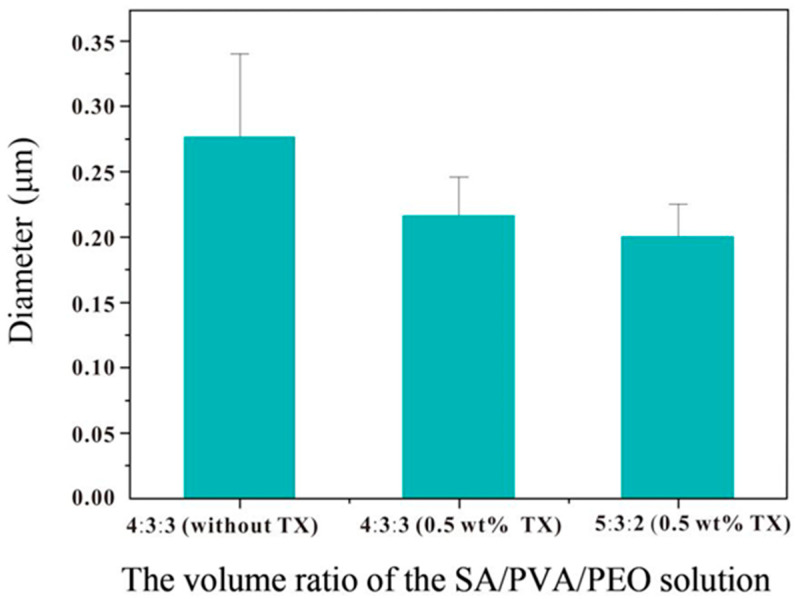
Average diameter distribution of three types of MFMs (TX: Triton X-100).

Other than morphological observations, an investigation on the effects of different materials on microfibers’ diameter was performed. The diameter of SA/PVA/PEO 4:3:3 microfibers with Triton X-100 is reduced from 276 ± 64.6 nm to 216 ± 30.24 nm, compared to that without Triton X-100, as shown in Figure 4. Meanwhile, the fiber diameter unevenness is significantly reduced, and the coefficient of variation of fiber diameter is reduced from 23.4 to 14.0%. Therefore, the addition of surfactants can improve the uniformity of microfibers, subsequently improving the quality of microfibers [60]. Nevertheless, as revealed in Figure 3, with 0.5 wt.% Triton X-100, SA/PVA/PEO 5:3:2 produces smaller microfiber diameters than SA/PVA/PEO 4:3:3, which is due to the decreased mass fraction of PEO and reduced chain entanglement.

### 3.2. Thermal Treatment to SA/PVA/PEO Microfiber Membranes

**Figure 5:**
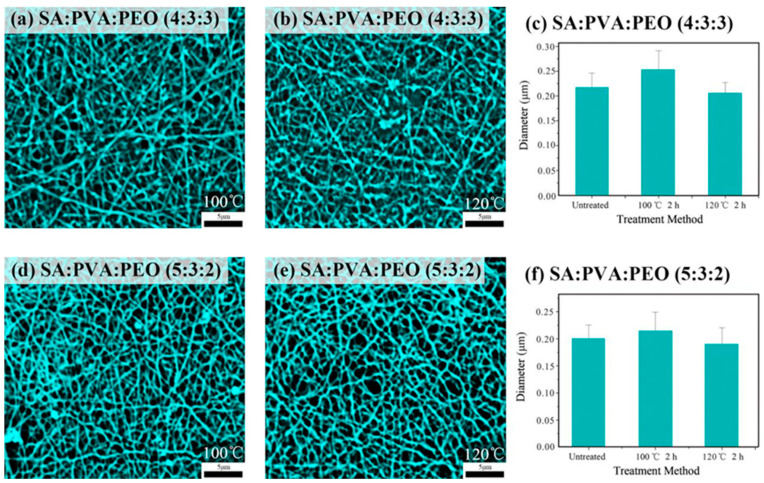
Surface morphological structure of SA/PVA/PEO 4:3:3 MFMs treated at (**a**) 100 °C and (**b**) 120 °C. (**c**) Average diameter of SA/PVA/PEO 4:3:3 MFMs treated at different temperatures. Surface morphological structure of SA/PVA/PEO 5:3:2 MFMs treated at (**d**) 100 °C and (**e**) 120 °C. (**f**) Average diameter of SA/PVA/PEO 5:3:2 MFMs treated at different temperatures.

Figure 5 shows the SEM morphology and fiber diameter distribution of thermally treated SA/PVA/PEO microfibers at different ratios of 4:3:3 and 5:3:2. It can be observed in Figure 5 that all MFMs showed different degrees of melting adhesion due to the melted PEO. When the microfibers of SA/PVA/PEO 4:3:3 and 5:3:2 were treated at 100 °C for 2 h, the diameter substantially increased compared to the untreated. However, as the temperature rises to 120 °C with a similar duration, the fiber diameter noticeably reduced. It is believed that the PEO in MFMs melted and flowed out slowly at 100 °C [34,61,62], most of which are glued to the outer layer of microfibers, and some might have flowed into the voids of the fibers. After thermal treatment, the PEO re-solidified and caused a substantial increase in microfibers’ diameters [63]. Meanwhile, at 120 °C, the PEO was prone to decompose and be volatilized [64], which eventually decreased fibers’ diameters compared with before treatment and the microfibers treated at 100 °C.

**Figure 6:**
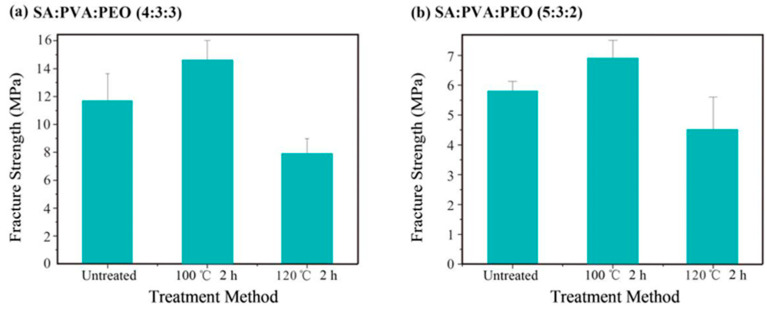
(**a**) Fracture strength of SA/PVA/PEO 4:3:3 MFMs treated at different temperatures (**b**) Fracture strength of SA/PVA/PEO 5:3:2 MFMs treated at different temperatures.

As shown in Figure 6a,b, trend changes are similar to what is revealed in Figure 5c,f. The average fracture strength of the untreated SA/PVA/PEO 4:3:3 MFMs is 11.7 MPa. After being thermally treated at 100 °C, point bonding occurred between the fibers due to the solidified melted PEO, which eventually improved the fracture strength of the MFMs. However, when the treatment temperature was increased to 120 °C, the fracture strength of the MFMs decreased due to the thermal decomposition and volatilization of PEO. Similarly, the fracture strength of SA/PVA/PEO 5:3:2 MFMs treated at 100 °C is greatly improved. Furthermore, changes in diameter size between untreated and treated MFMs showed great influence on the fracture strength of MFMs. As the diameter increases, the fracture strength will be increased too. This intertwined coincidence suggests that, to some extent, with the presence of PEO and a suitable temperature, improvements of fracture strength are feasible.

### 3.3. Antibacterial Efficacy of Berberine-Loaded SA/PVA/PEO Microfiber Membranes

Table 3 shows the antibacterial properties of MFMs containing different weight fractions of berberine (3 and 5 wt.%) against *E. coli*. Without berberine, the antibacterial rate of MFMs was only 14.7%, indicating a non-antibacterial characteristic. When 3 and 5 wt.% berberine were added, the inhibition rates of the MFMs against *E. coli* were 92.9 and 97.2%, respectively. Therefore, it can be deduced that berberine exhibited substantial antibacterial activity [41].

### 3.4. Water Dissolution Resistance of Berberine-Loaded SA/PVA/PEO Microfiber Membranes

**Figure 7:**
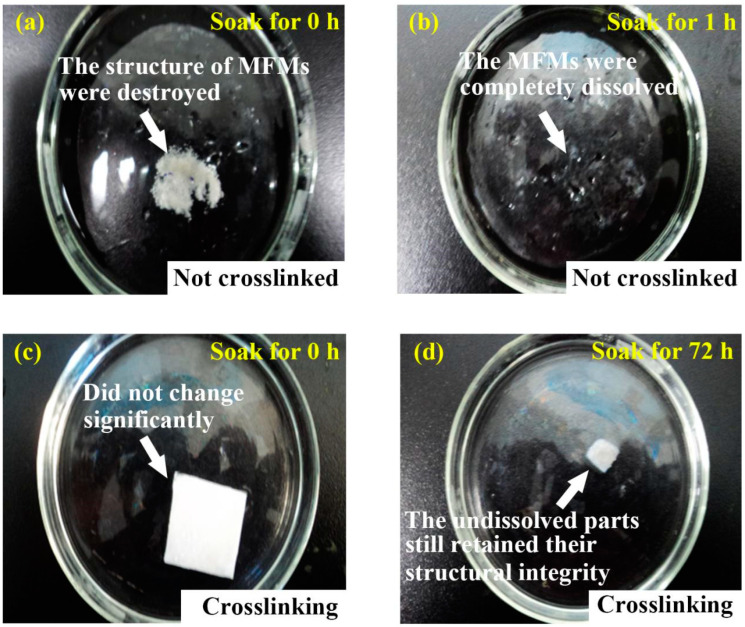
Water dissolution resistance of non-crosslinked SA/PVA/PEO 5:3:2 MFMs (**a**) soaked for 0 h, and (**b**) soaked for 1 h. Water dissolution resistance of crosslinked SA/PVA/PEO 5:3:2 MFMs with CaCl_2_ (**c**) soaked for 0 h, and (**d**) soaked for 72 h.

Figure 7 shows the water dissolution resistance of the MFMs before and after crosslinking treatment. It can be seen from Figure 7a that, upon soaking, the structure of non-crosslinked MFMs is clearly destroyed. Within 1 h, the MFMs are wholly dissolved, as seen in Figure 7b. However, as seen in Figure 7c, the crosslinked MFMs with CaCl_2_ structurally do not change upon soaking in water. Then, after about 72 h, as seen in Figure 7d, most of the crosslinked MFMs are dissolved and the surface area decreased by more than 80%. The undissolved parts of crosslinked MFMs still preserved their structural integrity. The reason is that SA can be crosslinked with most multivalent cations, which leads to reticular structure and reduces the degree of freedom of SA polymer chains. Ca^2+^ is crosslinked between the polymer chains and generated a three-dimensional structure to inhibit the flow of free water molecules. Therefore, the water solubility of MFMs is reduced, and the water dissolution resistance is increased [65,66,67].

### 3.5. Moisture Absorption and Fracture Strength of Berberine-Loaded SA/PVA/PEO Microfiber Membranes

**Figure 8:**
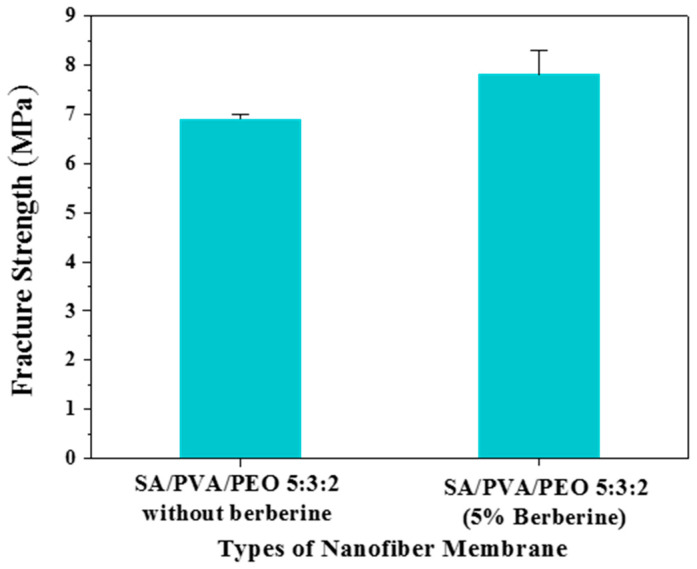
Fracture strength comparison between SA/PVA/PEO MFMs 5:3:2 without berberine and SA/PVA/PEO containing 5 wt.% berberine.

Cells cannot live without water, and microfiber wound dressings should have a certain moisture content to meet the requirement of wound cells’ growth environment [53]. The ideal wound dressing should be able to maintain a high moisture content [68]. The moisture content of MFMs with 5 wt.% berberine is 71.98%. The moisture content of the MFMs implies that it could maintain the moist environment for cell growth and promote cell growth and reproduction. Ass can be seen from Figure 8, the fracture strength of SA/PVA/PEO MFMs containing 5 wt.% berberine reached 7.8 MPa, which is higher than that of SA/PVA/PEO MFMs 5:3:2 without berberine. Berberine is a quaternary ammonium salt with considerable molecular weight and hard skeleton, enhancing the interaction between the polymer side chains and improving the mechanical properties of MFMs [69].

## 4. Conclusions

The addition of PEO improved the electrostatic spinning performance of SA and the mechanical performance of MFMs. With the addition of 0.5 wt.% Trition X-100, the proportion of SA solution in the spinning system has increased up to 50%. The SA/PVA/PEO 5:3:2 MFMs showed good fiber morphology with no clogging formation during spinning. The fracture strength of the MFMs was improved substantially by thermal treatment at 100 °C for 2 h. When different weight fractions of berberine, 3 and 5 wt.%, were added to SA/PVA/PEO MFMs, an excellent antibacterial property was observed, as well as decent moisture content, improved structural strength, and good water dissolution resistance. Berberine-loaded SA/PVA/PEO MFMs have the potential to be a new type of antibacterial wound dressing substrate with values that should be considered in the wound dressing field.

## Figures and Tables

**Table 1:** Thermal treatment of SA/PVA/PEO MFMs.

The Ratio of SA/PVA/PEO	Processing Temperature (°C)	Processing Time (h)
4:3:3	100	2
4:3:3	120	2
5:3:2	100	2
5:3:2	120	2

**Table 2:** Mixing ratios of 1 L PBS buffer.

Serial Number	Chemicals	Weight (g)
1	NaCl	8.0
2	KCl	0.2
3	Na_2_HPO_4_·12H_2_O	1.96
4	KH_2_PO_4_	0.24

**Table 3:** Bacteriostatic rates of SA/PVA/PEO 5:3:2 MFMs containing different mass fractions of berberine.

Sample Name	Bacterial Colony Count after Antibacterial (cfu·mL^−1^)	Bacteriostatic Rate (%)
Control sample	394	-
SA MFMs with 0% berberine	336	14.7
SA MFMs with 3% berberine	28	92.9
SA MFMs with 5% berberine	11	97.2

## Data Availability

Not applicable.
